# Preparation and characterization of a novel triple composite scaffold containing silk fibroin, chitosan, extracellular matrix and the mechanism of Akt/FoxO signaling pathway in colonic cancer cells cultured in 3D

**DOI:** 10.3389/fbioe.2023.1139649

**Published:** 2023-05-03

**Authors:** Zhipeng Cao, Liang Chen, Gengming Niu, Yan Li, Zhiqing Hu, Runqi Hong, Xiaotian Zhang, Liang Hong, Shanliang Han, Chongwei Ke

**Affiliations:** Department of General Surgery, Fifth People’s Hospital of Shanghai, Fudan University, Shanghai, China

**Keywords:** extracellular matrix, silk fibroin, chitosan, scaffolds, Akt/FoxO signaling pathway

## Abstract

This work examined the physical and chemical properties and biocompatibility *in vivo* and *in vitro* of a unique triple composite scaffold incorporating silk fibroin, chitosan, and extracellular matrix. The materials were blended, cross-linked, and freeze-dried to create a composite scaffold of silk fibroin/chitosan/colon extracellular matrix (SF/CTS/CEM) with varying CEM contents. The SF/CTS/CEM (1:1:1) scaffold demonstrated the preferable shape, outstanding porosity, favorable connectivity, good moisture absorption, and acceptable and controlled swelling and degradation properties. Additionally, HCT-116 cells cultivated with SF/CTS/CEM (1:1:1) showed excellent proliferation capacity, cell malignancy, and delayed apoptosis, according to the *in vitro* cytocompatibility examination. We also examined the PI3K/PDK1/Akt/FoxO signaling pathway and discovered that cell culture using a SF/CTS/CEM (1:1:1) scaffold may prevent cell death by phosphorylating Akt and suppressing FoxO expression. Our findings demonstrate the potential of the SF/CTS/CEM (1:1:1) scaffold as an experimental model for colonic cancer cell culture and for replicating the three-dimensional *in vivo* cell growth environment.

## 1 Introduction

With over 940,000 annual deaths, colonic carcinoma (CC) is the second most lethal malignant tumor around the world, followed by lung cancer (Sung et al., 2021). Due to their socioeconomic development, developed countries have the highest incidence of this disease (Dekker et al., 2019). The occurrence and development of tumors are multi-stage processes involving multiple biological pathways, and the mechanisms involved are relatively complex (Zhang et al., 2018). By combining the construction of the tumor environment with the study of the biological behavior of tumors and utilizing new research technologies and methods, greater understanding and progress have been made in tumor research in recent years. Therefore, developing novel tumor models and advancing tumor research are quite important.

These studies suggest that tumor growth has its own internal microenvironment. Cells are encapsulated by the outer matrix, and there are dynamic interactions between cells and the matrix and between cells and signal molecules. With different methods of building a tumor microenvironment, the goal is to simulate a microenvironment closer to the growth of tumors *in vivo* and facilitate biological behavior that is closer to real tumors *in vivo*. At this stage, biological research on tumors is mainly carried out at the level of two-dimensional (2D) single-cell culture. However, 2D culture has some limitations in the study of biological behavior and drug sensitivity of colorectal cancer, such as the reduction of tumor malignancy and changes in cell-related properties, such as differentiation and interaction between polarity and extracellular matrix (ECM), which cannot represent the real tumor focus. It has been reported that monolayer cell culture differs from the *in vivo* cells in terms of tumor drug mechanism and resistance, whereas various physiological activities of colon cancer cells in a three-dimensional culture environment are closer to the real environment *in vivo* (Shin et al., 2018). Cells in a three-dimensional (3D) culture system may retain a healthy proliferative state for a longer amount of time compared to 2D culture conditions, and cell activity is greatly increased. Therefore, we established previously a 3D tumor model for tumor drug screening (Lovitt et al., 2018). Although 3D scaffold materials are widely used in tissue engineering, there are few reports in the field of tumors, especially in colon cancer (Liang et al., 2020). Tissue engineering materials are creatively used to seed specific tumor cells into certain biomaterials, form cell biomaterial composites after *in vitro* culture, and implant them into mice to investigate the similarities and differences between tumor characteristics and traditional culture methods (Xie et al., 2016). In a 3D model, cells continue to proliferate and secrete matrix. At the same time, 3D materials are gradually absorbed. The final tumor tissue demonstrates significant differences in morphology, function, and other aspects of the tumor tissue with simple subcutaneous tumorigenesis. Therefore, some scholars have proposed that a 3D structure constructed *in vitro* should be fully used to simulate the tumor microenvironment (Landberg et al., 2020).

In three-position scaffold materials, the use of high-molecular-weight substances such as silk fibroin (SF) and chitosan (CTS) has been widespread. SF has a wide range of sources and good biological properties (Algarrahi et al., 2018; Galvez Alegria et al., 2019); thus, its increasing use in tissue engineering and has good prospects. The fast rate of breakdown of SF, however, limits its use (Chomchalao et al., 2013). As a linear polysaccharide with naturally occurring positively charged bases, CTS has excellent biofunctionality, plasticity, biodegradability, and safety (Zhao et al., 2020). A CTS 3D scaffold can provide space for cells to grow, multiply, and finally approximate the characteristics of organs with certain functions, which can then be used to study tumor tissues (Patrulea et al., 2015). Other components, including elastin, collagen, glycoproteins, and proteoglycans, can be found in the ECM. According to previous studies, the ECM can have an impact on a cell’s basic functions, including cell division, proliferation, adhesion, and phenotypic expression. In addition to help organizing tissues, it also provides crucial biochemical and biomechanical cues for regulating vascular and immune development, cell proliferation, migration, and differentiation. Furthermore, the ECM can control how dynamically cancers develop.

Researchers have been exploring composite systems with diverse polymers to overcome the drawbacks of scaffolds made of a single material. Combinations of various polymers are expected to confer their individual properties and form scaffolds that may promote cell adhesion, proliferation, and differentiation (Ghosh et al., 2019). Moreover, composite material scaffolds have been used and studied for cartilage repair (Bhardwaj et al., 2011), sciatic space repair (Gu et al., 2014), and bone defect reconstruction (Ríos et al., 2009).

This paper presents a new study on CC 3D culture using SF/CTS/colon extracellular matrix (CEM) composite scaffolds *in vitro*. For the first time, we investigated the creation of composite scaffolds based on the SF/CTS/CEM polymer system. We established a new 3D tumor cell culture system by seeding HCT-116 cells on SF/CTS/CEM scaffolds.

## 2 Materials and methods

### 2.1 Materials and animals

Genuine silkworm cocoons were obtained from farmers in Shiquan, Shanxi province. CTS powder (900,000 Da, 95% deacetylated), acetic acid, lithium bromide, dimethyl sulfoxide, absolute ethanol, cell counting kit (CCK)-8, dialysis bags, 4% paraformaldehyde, 1-Ethyl-3-(3-Dimethylaminopropyl) carbodiimide hydrochloride (EDC), and N-Hydroxysuccinimide (NHS) were purchased from Sangon Biotech (Shanghai) Co., Ltd., and sodium carbonate was acquired from the Sinopharm Chemical Reagent Co., Ltd. DMEM medium, 4,6-Diamidino-2-phenylindole dihydrochloride (DAPI), DY-554-Phalloidin staining, YF-488-Annexin V, and PI Apoptosis Kit were purchased from Share-bio Co., Ltd. Fetal bovine serum was purchased from Shrabio (Shanghai) Co., Ltd. Female naked mice aged 6 weeks were purchased from JSJ-lab (Shanghai) Co., Ltd. The mice were housed in a germ-free environment. The human CRC cell line HCT-116 was obtained from the Cell Bank of Shanghai Fifth People’s Hospital and cultured in DMEM medium.

#### 2.1.1 Extraction of silk fibroin and preparation of chitosan solution

The silkworm cocoon shell was cut into 1 cm^2^ pieces, immersed in 0.5% sodium carbonate solution, and boiled three times for 1 h each. It was then fully dried in an oven at 65 C after being rinsed three times in distilled water. The processed silk fibers were placed in a 9 M lithium bromide solution to form another solution, which was then dialyzed against distilled water (renewed every 12 h) in a dialysis bag (3.5 KD) for 72 h to obtain a 3% SF solution. To create a 3% CTS solution, CTS was dissolved in a solution of 3% glacial acetic acid.

#### 2.1.2 Preparation of colonic extracellular matrix

The colon tissue was soaked in 2% SDS+0.5% EDTA solution, which was changed every 3 h, shaken at room temperature for 12 h, and rinsed up with PBS buffer. The tissues were then immersed in 1% Triton X100 + 0.5% EDTA, which was changed every 3 h, shaken at room temperature for 12 h, and then rinsed up with PBS buffer to obtain the CEM. The CEM was ground into a powder by cold extraction with liquid nitrogen.

### 2.2 Scaffold synthesis and block design

As previously reported, chemical cross-linking techniques and freeze-drying technology were used to create the scaffolds used in the study (Li et al., 2017). SF scaffolds were made using only the 3% SF solution. SF and CTS were combined in a 1:1 (w/w) ratio to create SF/CTS (1:1) scaffolds. Three distinct mass ratios of the SF solution, CTS solution, and CEM powder were used to make the SF/CTS/CEM scaffolds with different ratios: 1:1:0.5, 1:1:1, and 1:1:2 (w/w). The mixture was then added to a solution containing 95% aqueous ethanol, 50 mmol/L EDC, and 18 mmol/L NHS, and the combination was agitated magnetically for 30 min to produce a homogenous solution. The solutions were then cast into well-sized 24-well and 96-well plates and crosslinked for 12 h at 4 C. The samples were then frozen at −20 C for 12 h and stored at −80 C for a further 12 h. To create the scaffolds, the samples were put into a freeze-dryer for 48 h. Before beginning cell culture, we used a low-temperature plasma sterilizer to decontaminate the scaffolds.

### 2.3 Characterization of the scaffolds

#### 2.3.1 Macroscopic appearance

Front and lateral views photographs were used to compare the various scaffold groups after they had been removed from the 24-well plate.

#### 2.3.2 Internal morphology

After removing the scaffolds from the 24-well place, a layer of scaffold film with the scaffold’s cross-sectional structure was left at the bottom of the well, and the structure of the scaffold film was examined under an optical microscope and captured on camera. The microstructures of the scaffolds were observed using a scanning electron microscope (SEM). The scaffolds were cut into small fragments with a surgical blade. We placed the prepared samples on the sample table and plated them with platinum. Then, the samples were put into the SEM and observed after vacuuming. We observed the microstructure and the microchannel of the scaffolds under the SEM and took photographs.

#### 2.3.3 Porosity evaluation

The liquid replacement method is used to assess scaffold’s porosity. Briefly, the scaffold is placed in anhydrous ethanol with a volume of V_0_. The volume of pure ethanol and the submerged scaffold after full immersion was V_1_. V_2_ was the volume remaining after the impregnated scaffold was removed. The following formula was used to determine porosity: (V_0_-V_2_)/(V_1_-V_2_) × 100%.

#### 2.3.4 Water uptake ratio

The mass of the scaffold in the dry state was recorded as W_1_. PBS buffer was used to rehydrate the dried scaffolds for 24 h at 37°C. With the aid of filter paper, any remaining liquid on the surface was removed, and the wet sample’s weight was recorded as W_2_. The following formula was used to compute water uptake ratio: (W_2_-W_1_)/W_1_×100%.

#### 2.3.5 Degradation property

The mass of the scaffold in a dry environment was W_0_. The scaffolds were placed in 6-well cell plate medium in PBS solution at 37 C, and then dried for 12 h at 65 C before being weighed at 1 d (day), 3 d, 7 d and 14 d (W_n_). The following formula was used to compute the degradation ratio: (W_0_–W_n_)/W_0_×100%.

### 2.4 Cell incorporation into scaffolds

Scaffolds were sterilized at low temperature in advance. We seeded 50,000 cells into each scaffold in 24-well plates, then slowly shook these plates, and finally added 1 mL of complete medium to each well. Every other day, the culture medium was changed.

#### 2.4.1 Cell adhesion in scaffolds

In a nutshell, HCT-116 105 cell (A_0_) cell suspensions were seeded on pre-wetted scaffolds, and adhesion rates were assessed 1, 3, and 6 h later. The scaffolds were taken out of the wells, and cells were counted (A_1_). Cells clinging to the well walls were digested and counted when the media was removed (A_2_). The following formula was used to calculate the cell adhesion rate: (A_0_-A_1_-A_2_)/A_0_×100%. Three experiments were performed for each condition, and the average adhesion rate was calculated.

#### 2.4.2 Cell proliferation in scaffold

CCK-8 was used to track cell proliferation in 2D plates, SF/CTS (1:1), and SF/CTS/CEM (1:1:1) scaffolds. Then, 100 μL of a cell suspension containing 10^3^ HCT-116 cells was seeded onto scaffolds in 96-well plates. Following the manufacturer’s instructions, proliferation of cell was assessed on 1 d, 3 d, and 5 d. Briefly, 10 μL CCK-8 reagent and 90 μL DMEM mixture was added to each well and the plate was placed for 90 min in the dark. Subsequently, we shocked the plates for 15 min and then the scaffolds were removed. To test absorbance at 450 nm, the remaining liquid was transferred to a fresh 96-well plate.

#### 2.4.3 Cell growth, micro-structure, and ultra-structure in scaffolds

##### 2.4.3.1 DAPI and DY-455-Phalloidin staining

HCT-116 cells (5 × 10^5^) were cultured in a pre-prepared scaffold. For cytoskeletal staining, 0.5% Triton X-100 in PBS was used to permeabilize the cells for 10 min at room temperature after cells being fixed with 4% paraformaldehyde for 15 min on ice. Using 10 g/mL, cytoskeletons were stained after incubation for 20 min at room temperature with a solution of DY-455-Phalloidin conjugates, then rinsed with PBS to get rid of any unbound DY-455-Phalloidin conjugates. Nuclear staining was performed with DAPI. A fluorescent microscope was used to record the images (Leica DM2500).

##### 2.4.3.2 Scanning electron microscope

HCT-116 cells were cultured on different groups of scaffolds. On days 1 and 3, the scaffold was washed with PBS for 3 times after the culture medium was removed. Then we used 4% paraformaldehyde to fix the cells. The plates were then placed in a refrigerator set at 80 °C for 12 h before being frozen at 20 C for another 12 h. The culture plates were then freeze-dried in a freeze-dryer for 48 h, and a SEM was used to examine the cell morphology on the scaffold.

##### 2.4.3.3 Hematoxylin-eosin staining

Hematoxylin and eosin (HE) staining was performed to observe biocompatibility of scaffolds *in vivo* and *in vitro*. On days 1 and 3 of scaffold culture, the composites were fixed with 4% paraformaldehyde. Meanwhile, 1 month after subcutaneous implantation of the scaffold containing cells, the nude mice were treated and the tumor was removed. HCT-116 cells and scaffolds from the removed tumors were fixed with 4% paraformaldehyde. After embedding in paraffin, the tissues were sectioned into slices of 3 μm thickness. Slices where then stained for HE and photographed with a Leica DM2500 microscope and an oil-immersion lens.

### 2.5 DY-488-Annexin V staining and flow cytometry assay

HCT-116 cells were seeded into different scaffolds and cultured in a 1 g/L low-glucose medium for 24 h to induce apoptosis. Cells were digested with trypsin without EDTA (ShareBio, Shanghai, China) and collected in Eppendorf tubes. Cells were stained using the Annexin V FITC Apoptosis Kit (ShareBio, Shanghai). A flow observation analyzer was used to assess the staining.

### 2.6 Protein extraction and Western blotting

A RIPA solution containing 1% PMSF was used to lyse the cells, and the entire protein extraction process was carried out on ice. Following that, protein samples were centrifuged for 15 min at 10,000 rpm at 4°C. Using the BCA reagent, protein concentrations were measured. Proteins were separated by electrophoresis on SDS gels and then transferred to PVDF membranes. Prior to being incubated with primary antibodies for 14 h at 4 C, the PDVF membranes were blocked with 5% nonfat milk for 1–2 h at room temperature. The secondary antibody was incubated for 1–2 h at room temperature after three membrane washes. After washing the secondary antibody solution from the membrane three times, protein expression was detected using an enhanced chemiluminescence reagent.

### 2.7 Immunohistochemical

Six-week-old mice were brought up in a pathogen-free environment. HCT-116 cells that had been conditioned in 2D, SF/CTS (1:1), and SF/CTS/CEM (1:1:1) were implanted subcutaneously into mice flanks. Using antibodies for Ki-67, PCNA, TUNEL, and Bcl-2 staining, tumor samples from mice were fixed in formalin and embedded in paraffin, sectioned into 5 μm slices, and subjected to immunohistochemical (IHC) staining. This was done 2 weeks and 1 month following cell implantation.

### 2.8 Statistical analysis

For all statistical calculations, the Graph Pad Prism 8 software was utilized. Each experiment was performed in triplicate. The mean and standard deviation (SD) were used to present quantitative data. The statistical significance was established using the Student’s t-test and analysis of variance (ANOVA). When differences were *p < 0.05, **p < 0.01, or ***p < 0.001, they were deemed significant.

## 3 Results

### 3.1 Characterization of the composite scaffolds in 3D

#### 3.1.1 Macroscopic appearance

**FIGURE 1:**
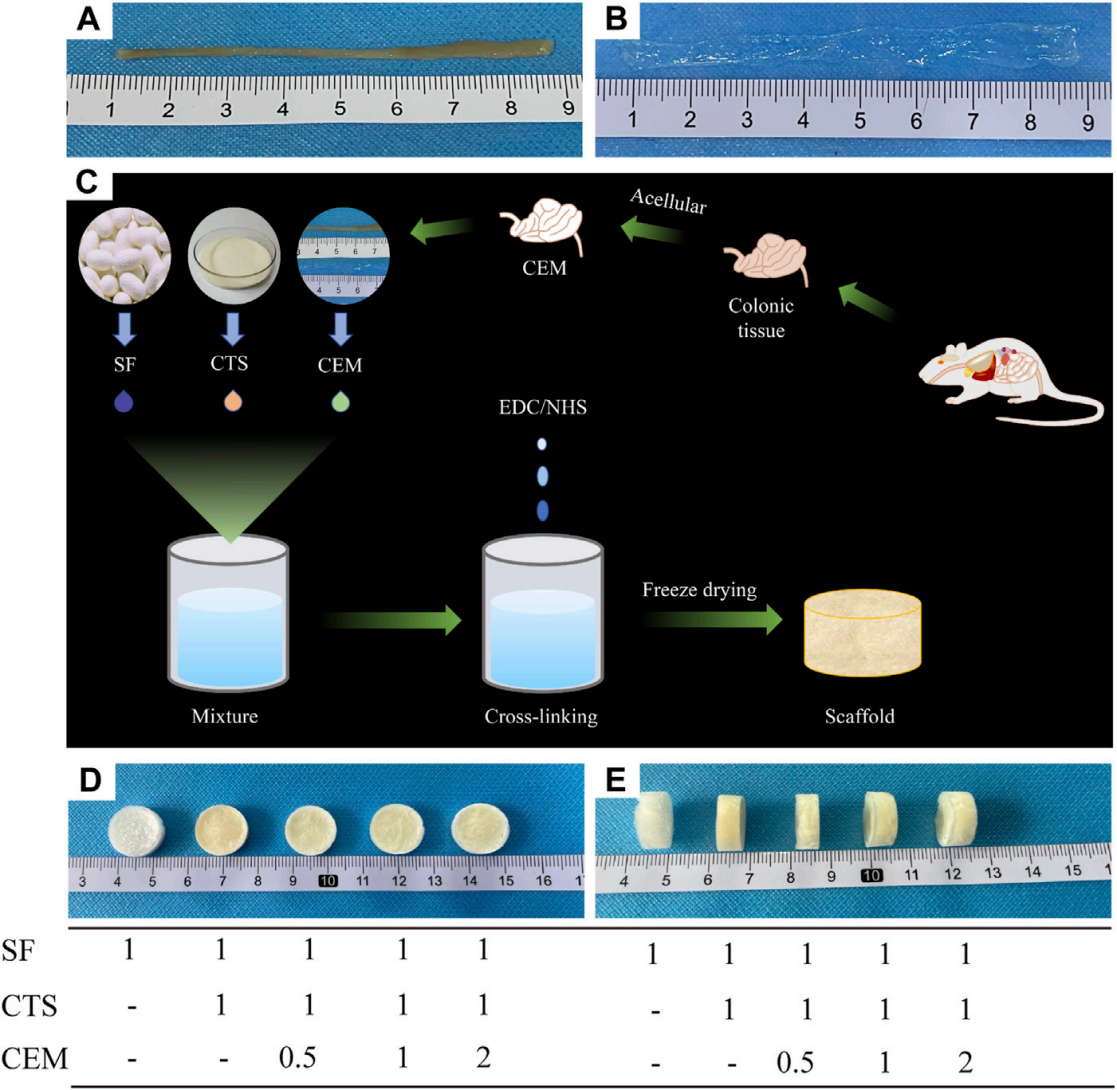
Colon of fresh nude mice and its extracellular matrix after acellular treatment **(A, B)**; Flowchart of the preparation of composite scaffolds by freeze-drying technique **(C)**; Front and side views of SF, SF/Cs (1: 1), SF/Cs/CEM (1: 1: 0.5), SF/Cs/CEM (1: 1: 1), and SF/Cs/CEM (1: 1: 2) scaffolds **(D, E)**. SF, silk fibroin; CTS, chitosan; CEM, colon extracellular matrix.

Figure 1 depicts the macroscopic characteristics of the SF, SF/CTS (1:1), SF/CTS/CEM (1:1:0.5), SF/CTS/CEM (1:1:1), and SF/CTS/CEM (1:1:2) scaffolds. We noticed that the shapes of all the scaffolds were comparable. Most of the scaffolds were yellowish-white in color, whereas the SF scaffold was completely white.

#### 3.1.2 Internal structure

**FIGURE 2:**
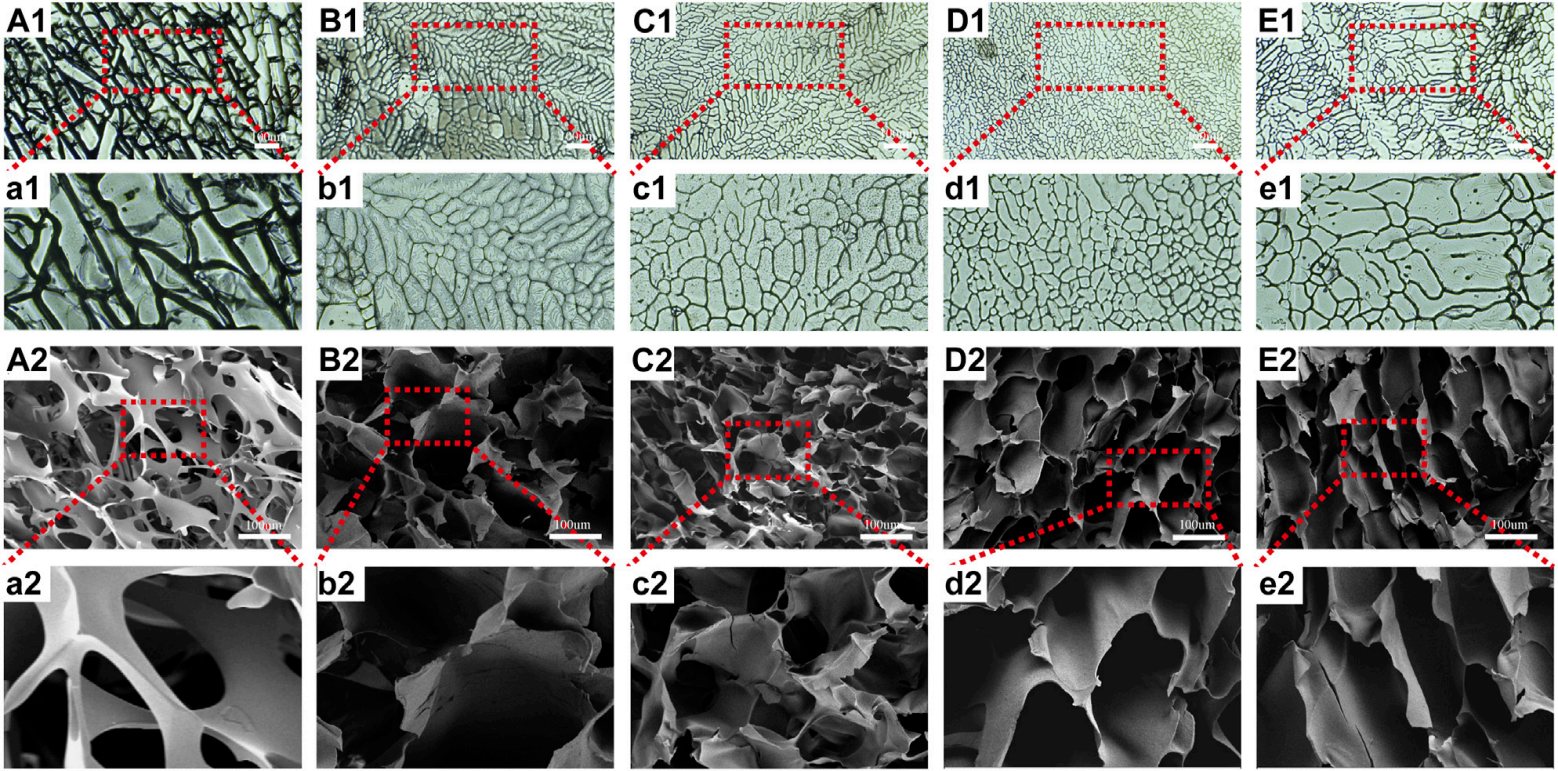
Images of optical microscope **(A1–E1, a1–e1)** and scanning electron microscope **(A2–E2, a2–e2)** of SF, SF/CTS (1: 1), SF/CTS/CEM (1: 1: 0.5), SF/CTS/CEM (1: 1: 1), and SF/CTS/CEM (1: 1: 2) scaffolds. SF, silk fibroin; CTS, chitosan; CEM, colon extracellular matrix.

To understand the internal structure of the scaffolds, we employed an optical microscope and SEM. As seen in Figure 2, every scaffold contained porous networks with architectures featuring various pore sizes, homogeneity, and strong pore connection. The addition of CTS and CEM changed the structure of pure SF scaffolds. The pore diameters and homogeneity of the SF/CTS/CEM (1:1:0.5), SF/CTS/CEM (1:1:1), and SF/CTS/CEM (1:1:2) scaffolds varied. As the proportion of CEM increased, the scaffold’s pore size steadily grew.

#### 3.1.3 Physical and chemical properties

##### 3.1.3.1 Porosity

**FIGURE 3:**
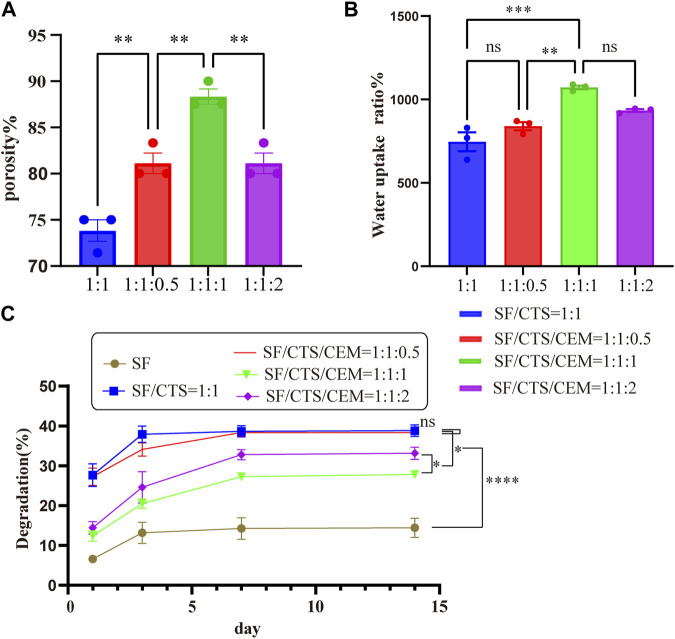
Results of porosity **(A)**, water uptake rate **(B)** and degradation rate **(C)** of different groups of scaffolds.*p < 0.05, **p < 0.01, ***p < 0.001.

All scaffolds had porosities greater than 68.69% (Figure 3A). Of all the scaffolds, the SF/CTS/CEM (1:1:1) scaffold had the highest porosity (88.33% ± 3.58%), followed by the SF/CTS/CEM (1:1:2) scaffold (81.11% ± 4.78%). The porosities of the SF/CTS and SF/CTS/CEM (1:1:0.5) scaffold were 81.11% ± 4.78% and 73.81% ± 5.12%, respectively. Our findings demonstrated that there was a statistically significant difference in the porosity of the SF/CTS (1:1) and SF/CTS/CEM (1:1:1) scaffolds (*p* < 0.05).

##### 3.1.3.2 Water uptake ratio

The water uptake ratios of the scaffolds are shown in Figure 3B. All of the scaffolds had water absorption rates in deionized water that were more than 501.9%. The maximum water uptake ratio was observed in the SF/CTS/CEM (1:1:1) scaffold (1,072% ± 48%), followed by the SF/CTS/CEM (1:1:2) scaffold (933.5% ± 36%). In contrast, the scaffold with the lowest water uptake ratio was the SF/CTS (1:1) scaffold (745.92% ± 44%). The results showed that there was significant difference in water absorption between SF/CTS (1:1) and SF/CTS/CEM (1:1:1) scaffolds.

##### 3.1.3.3 Degradation rates

Figure 3C displays the scaffolds’ degradation rates submerged in the PBS solution. The addition of CEM slowed the deterioration of the scaffolds. The SF/CTS/CEM (1:1:1) scaffold exhibited the lowest degradation rate, followed by the SF/CTS/CEM (1:1:2) scaffold. A significant difference observed between the two groups (*p* < 0.05). All five groups of scaffolds degraded to varying degrees within 14 days; however, the overall trends were similar. Day 3 was the turning point, as in the first 3 days degradation occurs rapidly, slowing down during the subsequent 4 days. From the 7th to 14th days, degradation stabilizes.

### 3.2 Cell incorporation into scaffolds

#### 3.2.1 Cell adhesion in scaffolds

**FIGURE 4:**
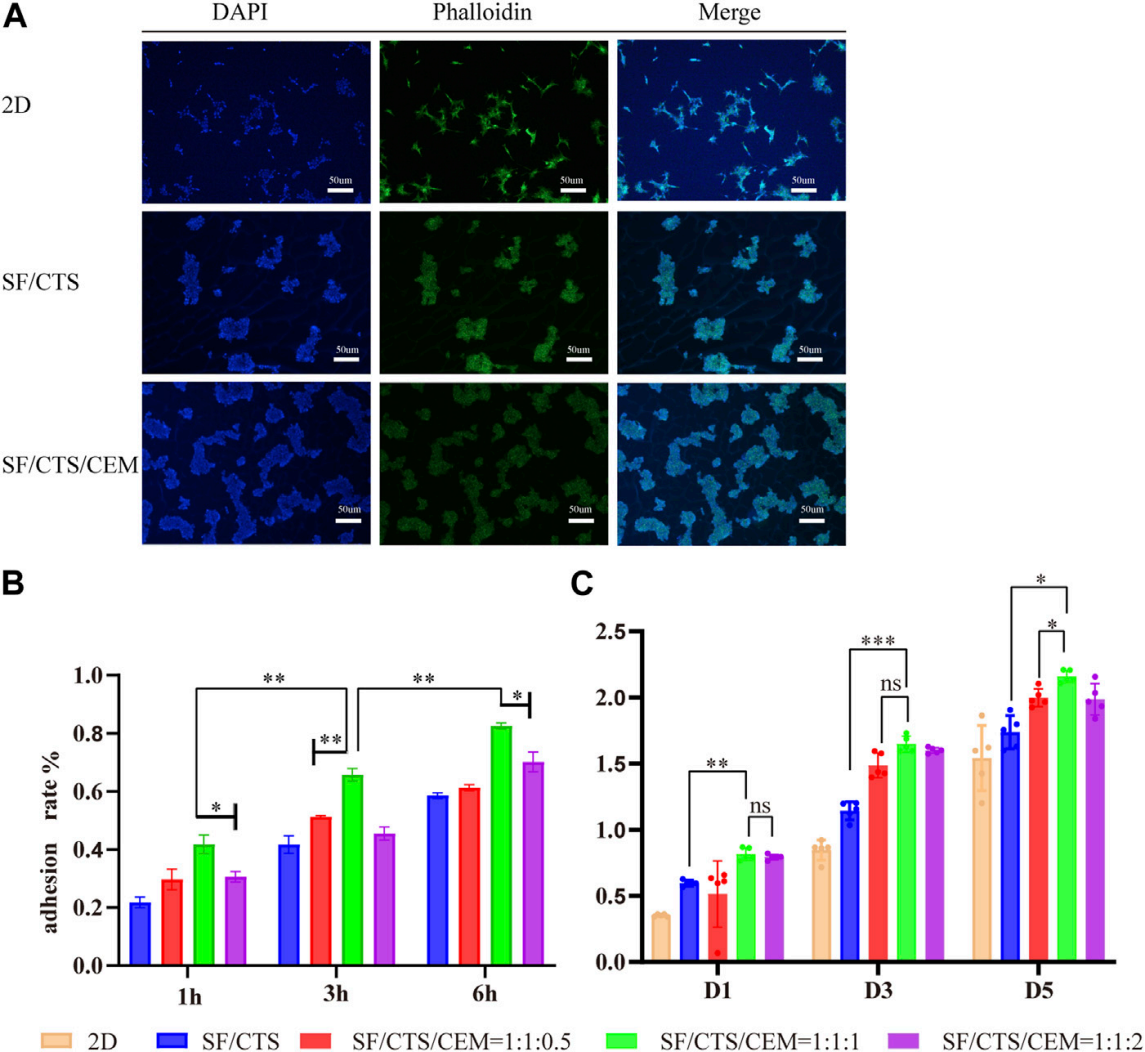
DAPI staining, Phalloidin staining and merging diagram of cultured HCT-116 cells in 2D, SF/CS (1:1) and SF/CS/CEM (1:1:1) **(A)**. Results of adhesion rate **(B)** and cell proliferation **(C)** of the different groups of scaffolds.*p < 0.05, **p < 0.01, ***p < 0.001. SF, silk fibroin; CTS, chitosan; CEM, colon extracellular matrix.

Counting cells on a cytometry plate at 1, 3, and 6 h after seeding the cells on the pre-wetted scaffolds allowed assessing cell adherence to the scaffolds. Figure 4B depicts the cell adhesion rate, showing that the SF/CTS/CEM (1:1:1) and SF/CTS/CEM (1:1:2) scaffolds had the highest rates. The SF/CTS/CEM (1:1:1) scaffold showed higher cell adhesion.

#### 3.2.2 Cell proliferation in scaffold

To gauge cell proliferation on the various scaffold groups, the CCK-8 kit was used (Figure 4C). According to our findings, the scaffold with the highest proportion of proliferating cells at each time point was the SF/CTS/CEM (1:1:1) scaffold. Based on the statistical findings, the cell proliferation of the two scaffolds was not significantly different on day 1, whereas the cell proliferation of the triple scaffold was significantly higher on days 3 and 5.

#### 3.2.3 Cell growth, micro-structure and ultra-structure in scaffolds

##### 3.2.3.1 DAPI and DY-488-Phalloidin staining

To visualize the cytoskeleton organization, we stained actin filaments with DY-488-Phalloidin-FITC and imaged the cells using a light microscope Leica DM2500 (Figure 4A). In 2D culture, the majority of the cells were long and fusiform, while the majority of the cells seeded in SF/CTS (1:1) and SF/CTS/CEM (1:1:1) scaffolds were spherical, which was more representative of the *in vivo* cell morphology. However, cell-cell attachments tended to be more pronounced in the SF/CTS/ECM (1:1:1) scaffold than in the SF/CTS (1:1) scaffold, with a spherical morphology. Additionally, we used DAPI to stain cell nuclei to confirm cell division, as shown in Figure 4A, which shows a statistically significant difference in the number of cells across the three scaffolds (2D, SF/CTS (1:1), and SF/CTS/CEM (1:1:1)) at all time points (*p* < 0.001). The proliferation ability of the SF/CTS/CEM (1:1:1) group was the highest.

##### 3.2.3.2 SEM

**FIGURE 5:**
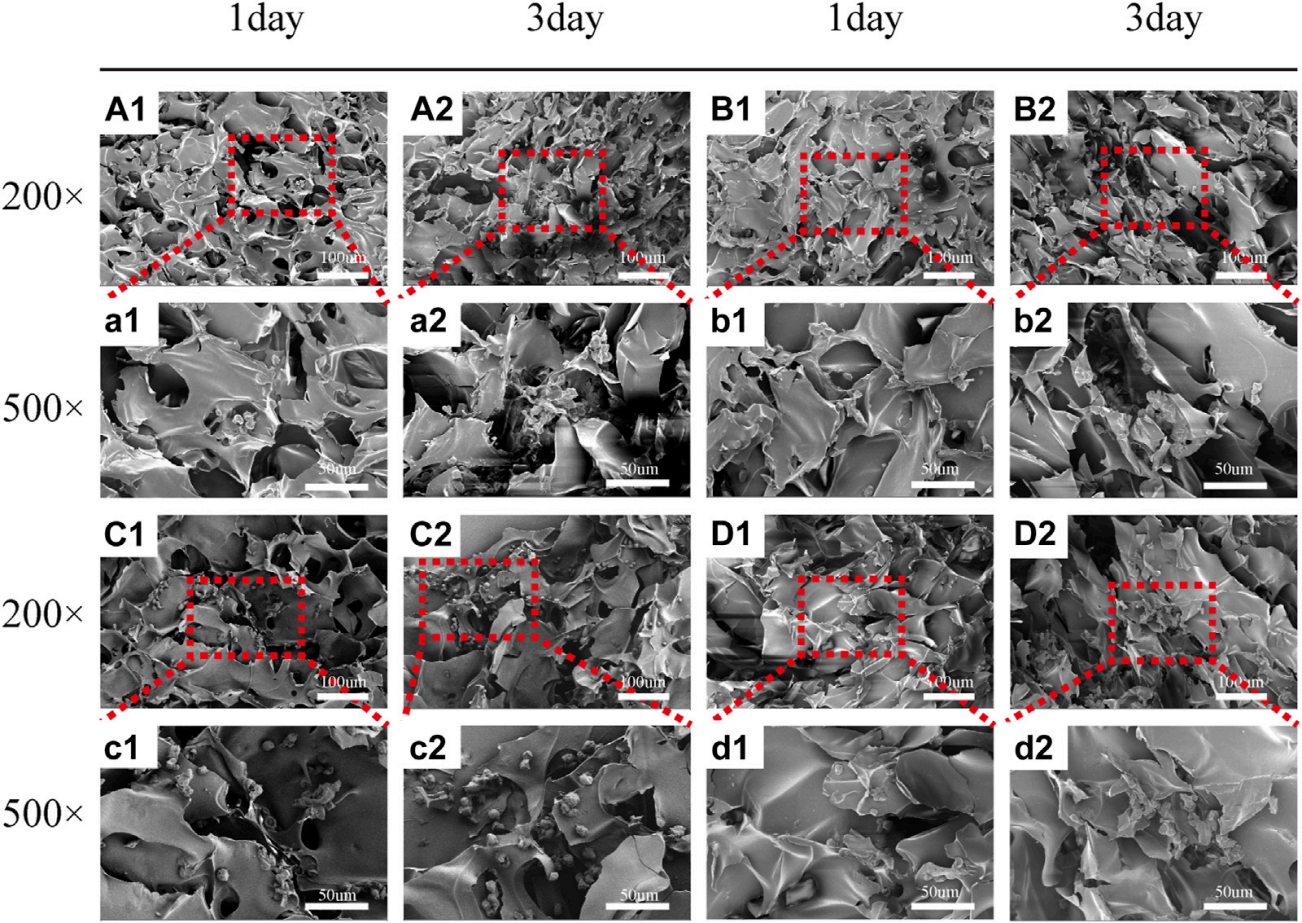
Scanning electron microscope images of HCT-116 cells on SF/CTS (1: 1), SF/CTS/CEM (1: 1: 0.5), SF/CTS/CEM (1: 1: 1), and SF/CTS/CEM (1: 1: 2) scaffolds. HCT-116 cells were cultured on different groups of scaffolds on day 1 **(A1–D1, a1–d1)** and day 3 **(A2–D2, a2–d2)**. SF, silk fibroin; CTS, chitosan; CEM, colon extracellular matrix.

Figure 5 depicts the SEM images of the cells on the scaffolds, displaying their morphology and health state. There was more room for cell division and proliferation in the SF/CTS/CEM (1:1:1) scaffold because the channel diameter was larger than that of the SF/CTS group (1:1). More cells proliferated on CEM-containing scaffolds. SF/CTS/CEM (1:1:1) scaffolds supported higher cell proliferation than SF/CTS (1:1) scaffolds under the same conditions. When compared to typical culture dishes, the cell morphologies in 3D scaffolds (SF/CTS/CEM (1:1:1) and SF/CTS (1:1)) were very different. In 3D scaffolds, the cells do not fully adhere to the scaffold and retain their body shape. The cells cultured in these scaffolds also had integrated spheres and, even though the number of cells grew over time, they did not spread across the inner surface of the scaffold. We also observed that the SF/CTS/CEM (1:1:1) scaffold supported a higher cell proliferation under the same conditions. The center portions of the scaffolds and the image are obtained (Figures 10 and 11). Figure 10 shows that internal situation of SF/CTS/CEM(1:1:1) scaffolds. Figure 11 shows the cell growth in the inner area of the scaffold with different material components. HCT-116 cells grow in the inner area of the different scaffolds. It was found that compared with the control group, the cells in SF/CTS/CEM (1:1:1) scaffolds group grew in lumps and had stronger proliferation ability.

##### 3.2.3.3 HE staining

**FIGURE 6:**
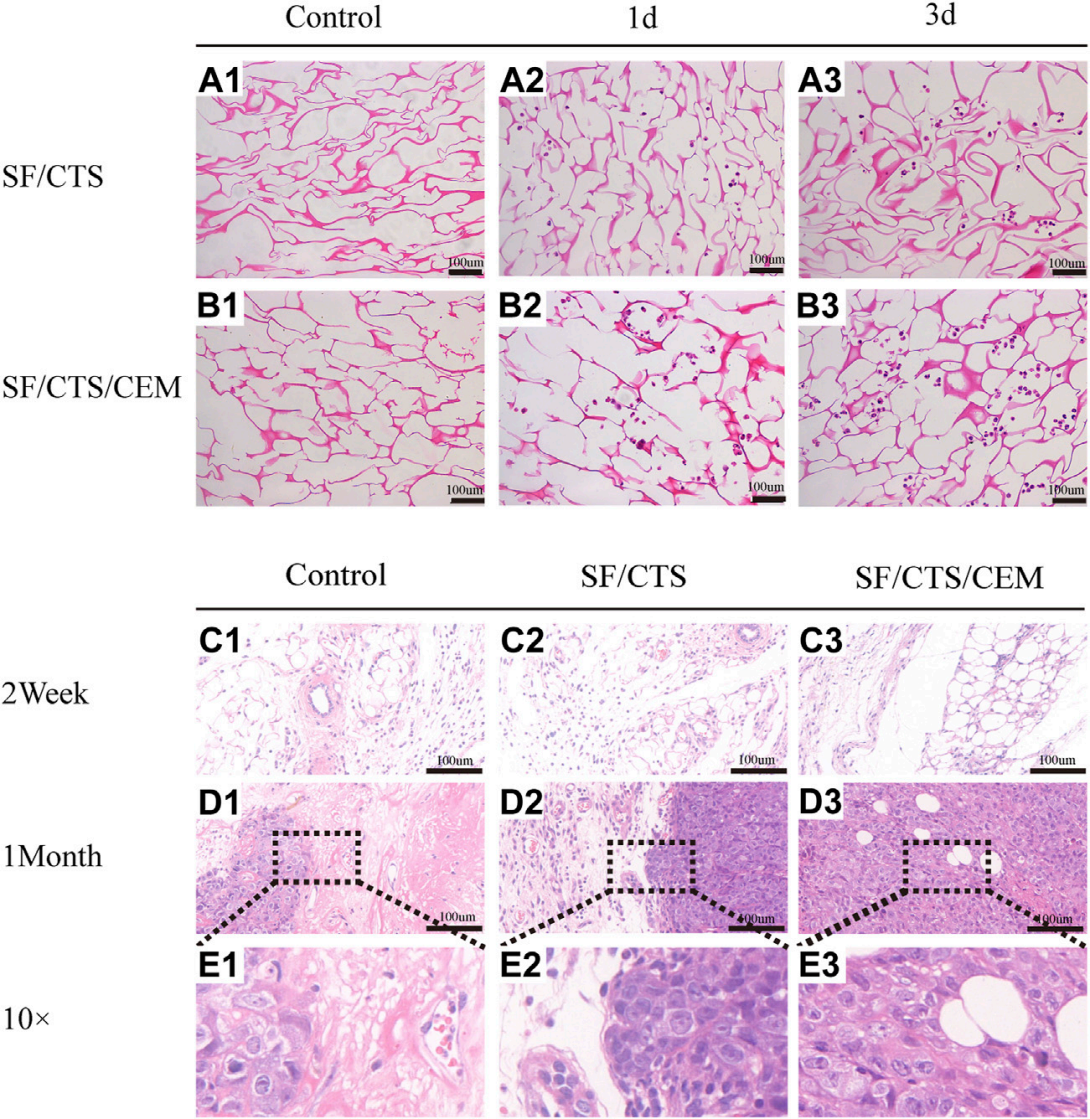
**(A)** Images of hematoxylin and eosin staining of scaffolds. Control groups were untreated SF/CTS (1:1) and SF/CTS/CEM (1:1:1) scaffolds, HCT-116 cells were cultured on SF/CTS (1:1) and SF/CTS/CEM (1:1:1) scaffolds on day 1 and day 3. **(B)** Images of hematoxylin and eosin staining of tumors formed after subcutaneous implantation of SF/CTS (1:1) and SF/CTS/CEM (1:1:1) scaffolds for 2 weeks and 1 month in nude mice. SF, silk fibroin; CTS, chitosan; CEM, colon extracellular matrix.

Images of HE-stained scaffolds are shown in Figure 6. Under the same conditions, the SF/CTS/ECM (1:1:1) scaffolds supported higher cell proliferation than the SF/CTS (1:1) counterparts. Compared with cells cultured in traditional 2D culture, cells on the scaffold tend to aggregate and grow. Notably, scaffolds implanted subcutaneously were gradually absorbed over time, and the tumor cells grew gradually. We also observed angiogenesis in the tumor.

### 3.3 Flow cytometry assay

**FIGURE 7:**
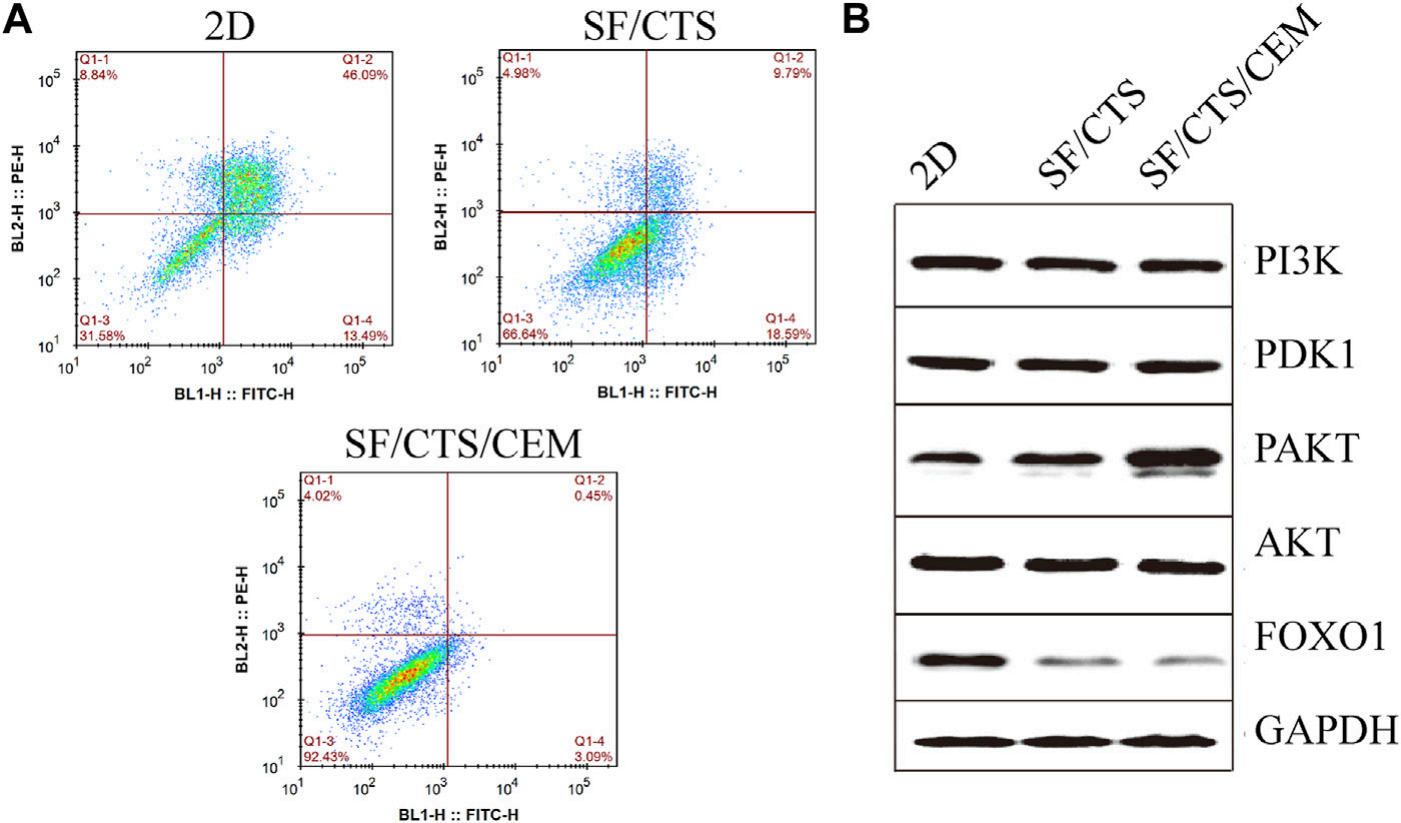
**(A)** Images of flow cytometry apoptosis of HCT-116 cells cultured in 2D, SF/CTS (1:1), and SF/CTS/CEM (1:1:1) scaffolds after apoptosis induction. **(B)** Analysis of protein expression of HCT-116 cells cultured on 2D, SF/CTS (1:1), and SF/CTS/CEM (1:1:1) scaffolds for 14 days. SF, silk fibroin; CTS, chitosan; CEM, colon extracellular matrix.

Flow cytometry analysis of apoptosis is shown in Figure 7. Under the same induction conditions, the apoptosis of HCT-116 cells cultured with the SF/CTS/CEM scaffold was the lowest, followed by the SF/CTS scaffold, with the highest apoptosis being observed in the 2D group.

### 3.4 Protein extraction and Western blotting

To confirm the influence of SF/CTS/CEM scaffolds on apoptosis, the PI3K/PDK1/Akt/FoxO signaling pathway was investigated by gel electrophoresis using protein samples of cells cultured for 14 days in the different conditions. SF/CTS/CEM (1:1:1) scaffolds activated Akt phosphorylation and inhibited the expression of pro-apoptotic FoxO compared with SF/CTS (1:1) and 2D cultures, as shown by protein expression (Figure 7).

### 3.5 IHC staining

**FIGURE 8:**
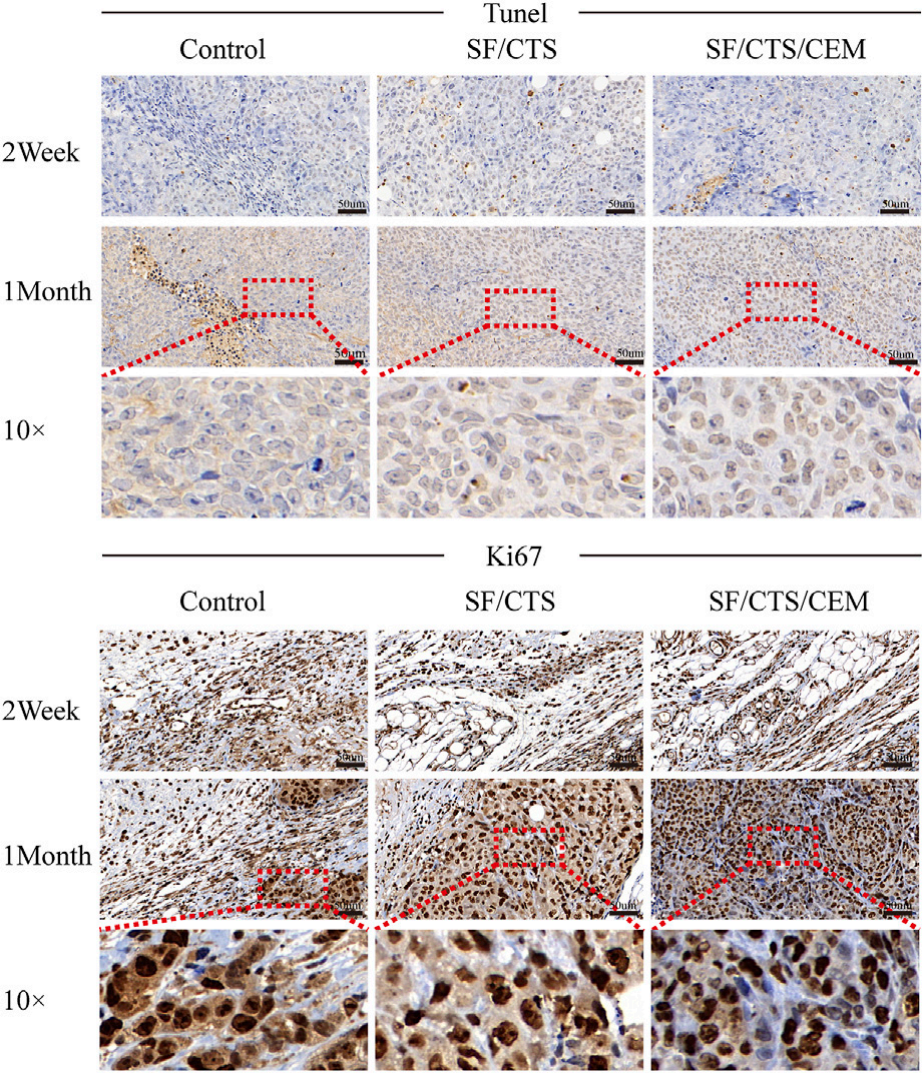
Immunohistochemical staining images of tumors formed by subcutaneous implantation of scaffolds. Two weeks and 1 month after implantation, tumor samples were subjected to immunohistochemical staining against TUNEL, and Ki67.

**FIGURE 9:**
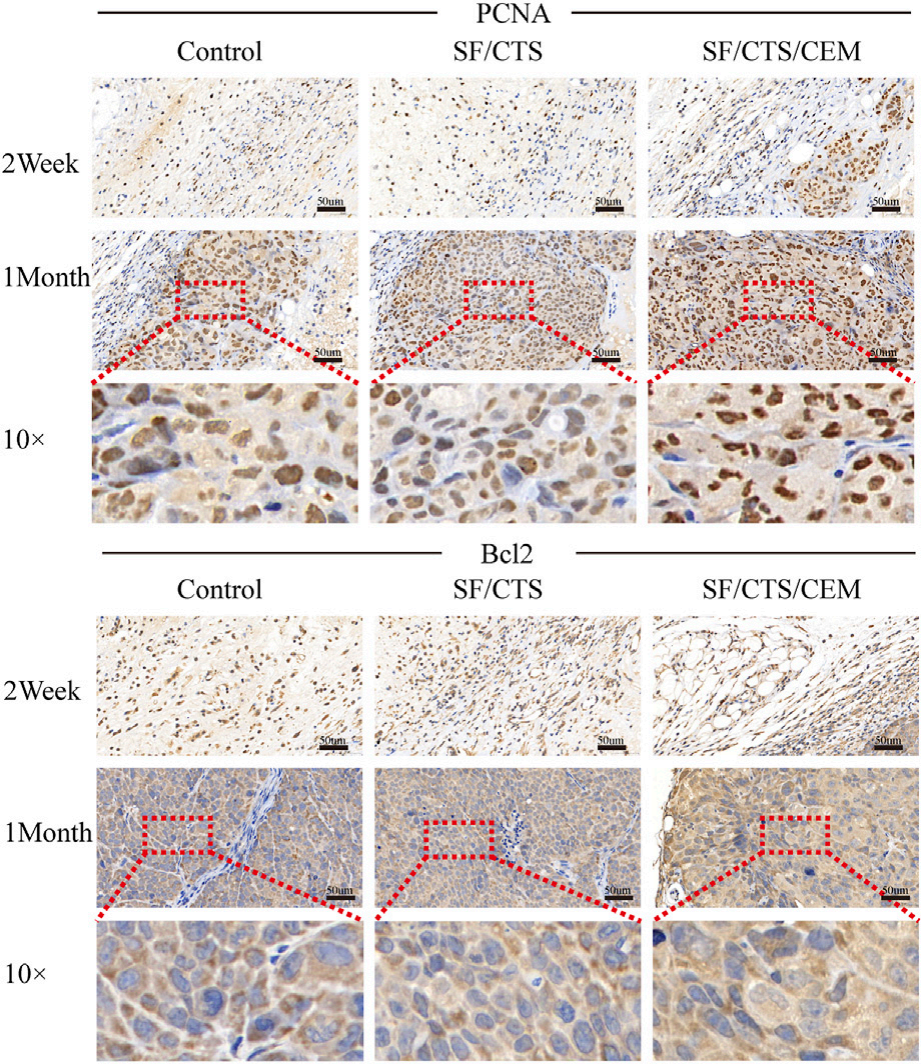
Immunohistochemical staining images of tumors formed by subcutaneous implantation of scaffolds. Two weeks and 1 month after implantation, tumor samples were subjected to immunohistochemical staining against PCNA and Bcl-2.

We subcutaneously injected HCT-116 cells grown on SF/CTS/CEM scaffolds or controls into the flanks of naked mice to determine whether tumor malignancy was reduced *in vivo* (n = 5 per scaffold). Figure 8 and Figure 9 illustrate how tumor cells cultured on SF/CTS/CEM scaffolds expressed Ki67 and PCNA at significantly higher levels than cells cultured in 2D. Bcl-2 and TUNEL expression were also downregulated.

## 4 Discussion

Conventional and classic 2D cell culture methods offer a practical platform for *in vitro* cancer. Cells cultivated on the surface of 2D flat Petri dishes, however, exhibit much less malignant phenotypes and do not accurately represent the same cell-ECM and cell-cell interactions as tumor formations *in vivo* (Smalley et al., 2006). To create scaffolds with the ideal culture properties, SF, CTS, and CEM have stimulated the development of 3D cell culture systems. Compared to SF scaffolds of pure silkworm, the blended scaffolds have different morphologies, porosities, elasticities, swelling behaviors, and biochemical compositions. Previously, the SF/CTS (1:1) mixture has been shown to be a potential biomaterial for the generation of scaffolds for cancer treatment (Gupta et al., 2009). In contrast to substrates made of pure biomaterials or synthetic polymers, according to a recent study 3D cell culture utilizing SF/CTS (1:1) scaffolds can promote cell proliferation in prostate cancer (Bäcker et al., 2017). Our preliminary experimental findings, however, indicated that SF/CTS (1:1) scaffolds had poor water absorption, a slow rate of breakdown, and a slow rate of cell adhesion, which restricts their use in 3D cell culture. Solid tumors are made up of genetically-mutated cancer cells surrounded by ECM and an additional group of genetically normal cells. The latter two elements are part of the tumor microenvironment and are important regulators of tumor biology, which has an impact on patients’ prognoses. The tumor ECM has been the focus of research for the past 20 years, exposing the fundamental biochemical and biological concepts and mechanisms underlying its function in tumor cell survival and proliferation. The ECM, however, also has a significant impact on immune cells in the microenvironment, controlling their differentiation and infiltration into tumor cells, as well as their proliferation and survival (Kolesnikoff et al., 2022). Currently, it is believed that the ECM chemical cues are the primary forces behind cancer formation and progression. Although the ECM mechanical forces have previously received little attention, they are now believed to be crucial to the development of illness and malignant cellular activity (Walker et al., 2018). The ECM surrounds the tumor cells, which interact dynamically with cytokines and signal transduction in its highly complicated milieu. The tumor microenvironment is a fertile ground for cell proliferation and malignant transformation, which can deter immune attacks, allow cancer cells to escape immune surveillance, cause surrounding infiltration and metastasis, and affect prognosis (Lu et al., 2020). We examined the changes in the biological features of cells following *in vivo* and *in vitro* culture using a 3D scaffolding method in order to evaluate the interaction between cells and the microenvironment (Liu et al., 2020). A 3D culture system was constructed, and preliminary exploration of the tumor microenvironment was performed (Wei et al., 2018). The study looked at how cells interact with the matrix using the biological framework of the TME that was built (Kievit et al., 2010; Mao et al., 2012). Based on the abovementioned advantages, to enhance the SF/CTS (1:1) composite scaffolds’ qualities and make them better suited for cell culture, we added CEM to them. Furthermore, the interior structure of the SF/CTS (1:1) scaffold is simpler than the tumor cell microenvironment *in vivo*, notably in terms of chemical composition, while being more complex than that of a 2D cell culture system. The scaffold’s chemical complexity can be increased, improving its ability to mimic the tumor milieu. This is accomplished by adding a CEM component. We predicted that the triple biomaterial composite scaffold would perform better than those consisting of just two biomaterials in terms of both features and performance. Our findings were intriguing in that HCT-116 cell spheres displayed tumor-like morphological characteristics seen *in vivo* in all four 3D scaffolds (SF/CTS (1:1), SF/CTS/CEM (1:1:0.5), SF/CTS/CEM (1:1:1), and SF/CTS/CEM (1:1:2)). The extremely aggressive activity typical of tumor cells *in vivo* was more likely to be displayed by cells growing in a 3D scaffold made of SF/CTS/CEM (1:1:1).

**FIGURE 10:**
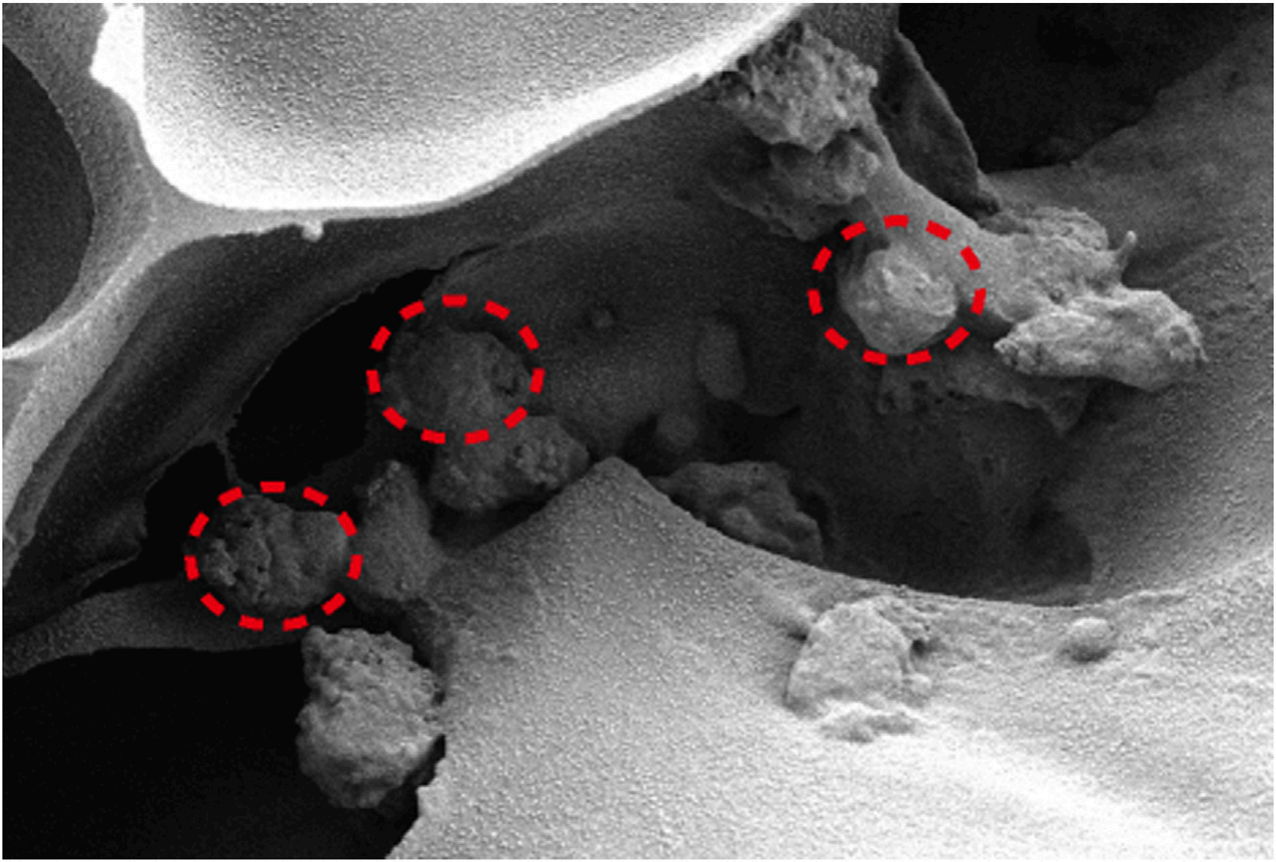
The interior environment of the SF/CTS/CEM(1:1:1) scaffolds can be seen in SEM images. The indicated region demonstrates how cells are expanding within the pores.

**FIGURE 11:**
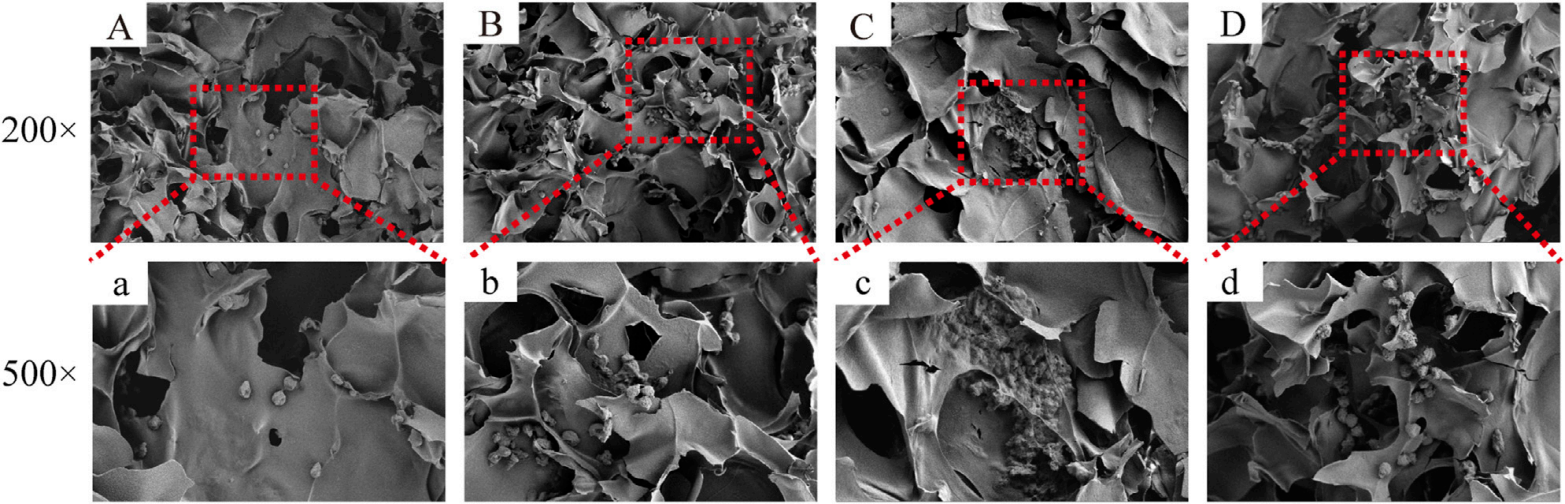
The figure shows the cell growth in the inner area of the scaffold with different material components. HCT-116 cells grow in the inner area of the SF/CS, SF/CTS/CEM(1:1:0.5), SF/CTS/CEM(1:1:1) and SF/CTS/CEM(1:1:2) scaffold **(A-D, a-d)**. Compared with **(A, a; B, b; D, d)**, the cells in SF/CTS/CEM (1:1:1) scaffolds group **(C, c)** grew in lumps and had stronger proliferation ability.

The Interaction between the OH- and COO- groups in SF, CTS, and CEM complexes is thought to produce interactions, enhancing the tensile strength of triple composite polymers. CEM might be added to the SF/CTS (1:1) composite scaffolds to increase their overall porosity and average pore diameter. Cells require physical room to function, which improves their ability to utilize nutrients and oxygen, as well as promoting effective removal of metabolic waste. These variables significantly affect the metabolic processes, cell attachment, proliferation, distribution, and differentiation (Nava et al., 2016). The optimal pore diameter depends on the specific cell type (Murphy et al., 2016). In our study, the SF/CTS/CEM (1:1:1) scaffold was the best for the culture and development of HCT-116 cells, due to its wide average pore width. High-porosity scaffolds improve mechanical interlocking and cell infiltration (Ionescu and Mauck, 2013). The SF/CTS/CEM (1:1:1) scaffold showed the best improvement of cell proliferation in our investigation and had the maximum porosity (88.33% ± 3.58%). For cell-infiltrating scaffolds, higher water absorption is preferred. A lower expansion rate helps maintain the structural stability of the scaffold. Here, a rise in the CEM percentage was associated with an increase in swelling and water absorption. According to our findings, the SF/CTS/CEM (1:1:1) scaffold exhibited a high water uptake ratio (1,072% ± 48%), was porous, and degraded at an optimum rate (Figure 3). Further characterization of the biological features of these scaffolds showed that the cells on the SF/CTS/CEM (1:1:1) scaffold exhibited the highest cell adhesion and proliferation rates (Figure 4), which supported the cell growth and morphological characteristics shown in the SEM images (Figure 5). The complete analysis of the biological features suggest that the optimal scaffold for the *in vitro* investigation of CC cells may be the SF/CTS/CEM (1:1:1) scaffold (Figure 10). Staining of the scaffold and cells revealed that the cells in the 3D scaffold kept their *in vivo* shape. Significant alterations in cell structure, protein expression, and mechanical properties occur during their migration through new tissues *in vivo* or in a new environment *in vitro*. It is well known that during movement, migration, adhesion, and proliferation, the cytoskeleton can undergo changes (Jin et al., 2012). In addition, cytoskeletal networks are essential for preserving cell shape (Ma et al., 2012). According to previous reports, a crucial condition for cell cycle progression is the cytoskeleton’s proper configuration (Jiang et al., 2013). According to our findings, the 2D culture cells developed the slowest, followed by those on the SF/CTS/CEM (1:1:1) and the SF/CTS (1:1) scaffolds. The majority of the cells in the 2D condition were long and fusiform, but the cells on the SF/CTS/CEM (1:1:1) and SF/CTS (1:1) scaffolds were round or nearly round (Figure 11). This indicated that the cells cultivated on scaffolds were more compatible with the growth status of cells *in vivo*. The HCT-116 cells transplanted on the 3D scaffold tended to assemble into multicellular spheres in contrast to the 2D monolayer model. These 3D multicellular aggregates serve as a useful alternative to traditional tumor models in cancer research (Hirschhaeuser et al., 2010). Our findings demonstrated that, compared to the SF/CTS (1:1) scaffold, the SF/CTS/CEM (1:1:1) scaffold supported sphere growth more significantly (Figure 5) and (Figure 11).

We also found that the cells cultured on SF/CTS/CEM (1:1:1) scaffolds showed significant proliferative ability, malignancy, and delayed tumor cell apoptosis compared with the those cultured in SF/CTS (1:1) scaffolds and 2D conditions. The PI3K/PDK/Akt/FoxO signaling pathway is a classical apoptosis regulator; therefore, we assessed the expression of this pathway. The findings demonstrated that p-Akt expression was upregulated and FoxO expression was downregulated in SF/CTS/CEM-cultured cells. These results suggest that the delay in apoptosis in scaffold culture is related to the PI3K/PDK/Akt/FoxO signal transduction pathway.

Despite the fact that our research sheds light on the development and possibility of fabricating composite scaffolds employing complimentary biopolymers, some issues still need to be addressed. As an example, the fundamental mechanisms observed in this study are still not well understood. In addition, in terms of porosity, water absorption, cell adhesion, proliferation, and sphere formation, our SF/CTS/CEM (1:1:1) scaffold performed well, although it came in second place in terms of disintegration rate. The association between the rate of degradation and cell proliferation cannot be inferred. Before these 3D models may be altered and widely used in medicine and basic science, further research is required to validate and clarify our findings and to better understand the potential mechanisms of cell growth and proliferation on these scaffolds.

## 5 Conclusion

The biological activity of CC cells differed depending on the type of material employed, as demonstrated by our analysis of the different HCT-116 cell culture conditions (2D, SF/CTS (1:1), and SF/CTS/CEM (1:1:1) scaffolds). According to our findings, the SF/CTS/CEM (1:1:1) scaffold has the potential to be a useful tumor model for CC cell culture studies and a realistic representation of cell growth in a 3D environment, as in living organisms.

## Data Availability

The original contributions presented in the study are included in the article/Supplementary Material, further inquiries can be directed to the corresponding authors.
