# Supplementary figures and images for: Preparation and characterization of a novel triple composite scaffold containing silk fibroin, chitosan, extracellular matrix and the mechanism of Akt/FoxO signaling pathway in colonic cancer cells cultured in 3D
Source: Front Bioeng Biotechnol. 2023 May 3;11:1139649. doi: 10.3389/fbioe.2023.1139649 (PMC10188982; doi:10.3389/fbioe.2023.1139649)

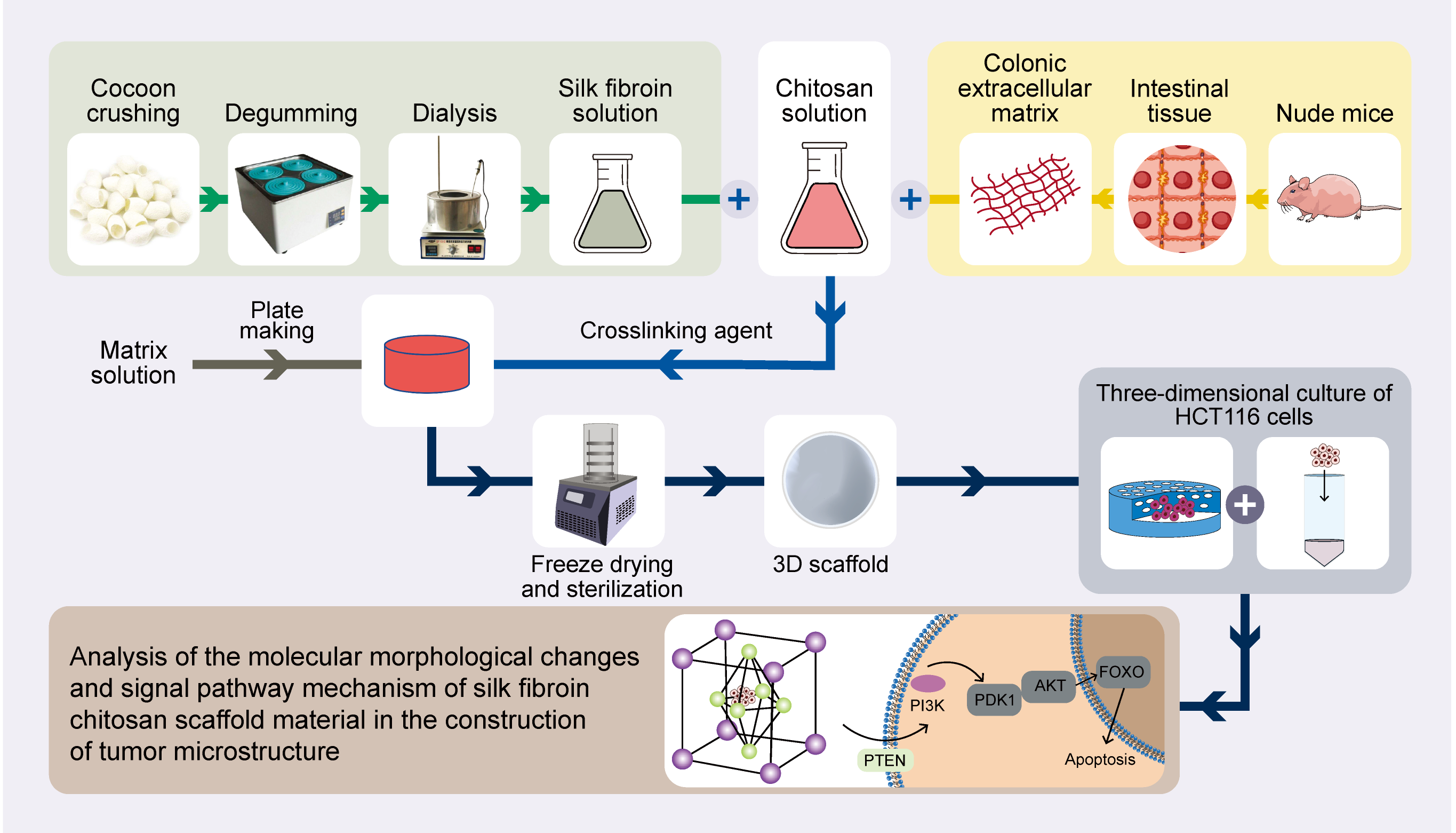

Supplement: Supplementary file 1 [file Image1.TIF]
